# Identification of potential hub genes related to ferroptosis and hypoxia in dilated cardiomyopathy: a bioinformatic analysis with preliminary experimental validation

**DOI:** 10.3389/ebm.2026.10709

**Published:** 2026-03-02

**Authors:** Xiqin Wang, Chrismis Novalinda Ginting, William Leslie

**Affiliations:** Medicine, Dentistry, and Health Science, Universitas Prima Indonesia, Medan, Indonesia

**Keywords:** dilated cardiomyopathy, ferroptosis, hypoxia, immune infiltration, lncRNA-miRNA-mRNA network

## Abstract

The study aims to explore the potential role of ferroptosis and hypoxia in dilated cardiomyopathy (DCM). GSE120895, GSE17800, GSE112556, ferroptosis-related genes (FRGs), and hypoxia-related genes (HRGs) were downloaded from the public dataset. Ferroptosis- and hypoxia-related differentially expressed genes (DEGs) and DCM-related genes were obtained. Subsequentially, hub genes were identified, and their diagnostic values were assessed. Next, immune cell infiltration analysis, drug prediction and molecular docking were carried out based on the hub genes. Finally, the hub gene TGM2 was preliminarily verified *in vitro*. A total of 18 ferroptosis- and hypoxia-related DEGs and 315 DCM-related genes were acquired. Subsequently, 6 hub genes (PPP1R15A, TGM2, MAP3K5, USP7, SESN2, and ADAM23) were obtained and have potential diagnostic value. Immune infiltration analysis showed that CD56dim natural killer (NK) cells, macrophages, monocytes, NK cells, and NK T cells were significantly infiltrated in DCM patients. Furthermore, the lncRNA-miRNA-mRNA network was constructed. Moreover, 16 drugs were predicted, and the binding energy between atorvastatin and TGM2 was −2.79 kcal/mol. *In vitro* verification showed that TGM2, PPP1R15A and SESN2 were up-regulated in DOX-induced AC16 cardiomyocyte injury. After knocking down TGM2, the expressions of α-actinin and cTnT were increased, and the expression level of HIF-1α was inhibited. Dual luciferase assay showed that hsa-miR-291-5p exerted its regulatory effect by directly binding to TGM2. Flow cytometry results showed that TGM2 had no significant effect on the apoptosis of AC16 cells. Our findings may provide new ideas for the diagnosis and treatment of DCM.

## Impact statement

DCM is a disease that primarily affects the myocardium, causing the heart to become dilated and reducing its ability to pump blood. As the condition progresses, heart function further deteriorates, potentially leading to heart failure, arrhythmias, or other complications. Recent evidence suggests that ferroptosis and hypoxia play important roles in cardiomyopathies. Our study employs bioinformatics methods to identify the hub genes related to ferroptosis and hypoxia in dilated cardiomyopathy. Through bioinformatical analysis, 6 hub genes were identified, and these genes exhibited better performance in distinguishing DCM from healthy controls. After knockdown TGM2, the expressions of α-actinin and cTnT were increased. Flow cytometry results showed that TGM2 had no significant effect on the apoptosis of AC16 cells. Our findings may provide new insights into understanding the role of ferroptosis and hypoxia in the development of DCM and may offer new treatment targets.

## Introduction

Dilated cardiomyopathy (DCM), a heart muscle disease, is defined as the dilation of the left ventricular (LV) or biventricular and impaired contractility [1]. DCM is a primary risk factor for developing heart failure (HF) and severe arrhythmia [2]. In clinical practice, echocardiography is employed to evaluate ventricular dilatation, cardiac magnetic resonance imaging is used to assess fibrosis and oedema, and myocardial biopsy is performed to examine inflammation. The pathophysiology of DCM is various, including genetic mutations, inflammation, autoimmunity, infections, and chemical and toxin exposure. However, the etiology and pathogenesis of DCM remain unclear and require further investigation.

Recent evidence suggests that ferroptosis plays an important role in cardiomyopathies [3, 4]. Ferroptosis, an iron-dependent form of non-apoptotic cell death, is caused by lipid peroxidation and reactive oxygen species (ROS) accumulation [5]. Fang et al. demonstrated that mitochondrial oxidative damage is a key mechanism underlying ferroptosis-induced cardiac injury in cardiomyopathy [6]. Additionally, hypoxia usually causes an increase in ROS and oxidative stress, which is strongly associated with cardiomyopathy [7, 8]. Intermittent hypoxia causes high blood pressure, LV remodeling, and dysfunction in rodent models [8]. Chick embryos exposed to hypoxia exhibited LV dilatation, decreased LV ejection fractions, and diastolic dysfunction [9]. In ischemia/reperfusion (I/R) injury myocardium, liproxstatin-1, a ferroptosis inhibitor, could decrease myocardial infarct sizes and mitochondrial ROS production to protect the myocardium [10]. Another ferroptosis inhibitor, dexrazoxane, impedes myocardial cell death caused by ROS and iron [11]. The above results indicate that ferroptosis and hypoxia were involved in cardiomyopathy, yet the potential role of ferroptosis and hypoxia in DCM remains unclear.

In this study, based on ferroptosis- and hypoxia-related genes, the hub genes in DCM were identified using weighted gene co-expression network analysis (WGCNA) and the least absolute shrinkage and selection operator (LASSO) regression analysis. Our findings may provide new insights into understanding the role of ferroptosis and hypoxia in the development of DCM and may offer new treatment targets.

## Materials and methods

### Data collection and preprocessing

GSE120895 (platform: GPL570) and GSE17800 (platform: GPL570) datasets were collected from the Gene Expression Omnibus (GEO) database^1^. The GSE120895 dataset contained 8 control samples and 47 cases of endomyocardial biopsies (EMBs) from DCM patients, which is used as the training set to identify hub genes. The GSE17800 included 8 control samples and 40 cases of EMBs from DCM patients, which is used as the validation set to verify hub genes. The “affy” (version 1.68.0) package in R was used for data processing. Gene expression data were normalized by Robust Multi-array Average (RMA) algorithm, which included background correction, quantile normalization, probe summarization and log2 transformation. All subsequent statistical analyses were performed based on the expression values after log2 transformation.

A total of 564 ferroptosis-related genes (FRGs) were acquired from the Ferroptosis database V2 (FerrDb,^2^). A total of 200 hypoxia-related genes (HRGs) were collected from the Molecular Signatures Database (MSigDB,^3^). All data and statistical analyses were performed in R software (version 4.0.5).

### Differential expression analysis and functional enrichment analysis

The DCM-related differential expressed genes (DEGs) with false discovery rate (FDR) <0.05 were identified in the GSE120895 dataset using the “limma” (version 3.46.0) package in R. Then, 564 FRGs, 200 HRGs, and DCM-related DEGs were intersected to obtain ferroptosis- and hypoxia-related DEGs. The volcano plot was used to visualize these results. Subsequently, Gene Ontology (GO) and Kyoto Encyclopedia of Genes and Genome (KEGG) functional enrichment analysis of ferroptosis- and hypoxia-related DEGs were carried out through the DAVID database^4^. The screening criterion for terms was *p*-value <0.05.

### WGCNA

The “WGCNA” package in R was utilized to perform WGCNA in the GSE120895. First, the “hclust” function was used to cluster all samples and detect the outliers. The high degree was set to 0.9, and the function “pickSoftThreshold” was utilized to calculate the soft threshold to construct a scale-free co-expression network. The weighted adjacency matrix was transferred into the topological overlap matrix (TOM) and 1-TOM. Genes with similar expression patterns were clustered, and modules were divided by default parameters according to the “cutreeDynamic” function. Since the modules identified by the dynamic tree cutting algorithm may be similar, these modules were merged at a height cutoff of 0.25. The “moduleEigengenes” function was used to calculate module eigengene (ME) and Pearson analysis was employed to identify DCM-related ME. Genes with |gene significance (GS)| >0.4 and |module membership (MM)| >0.4 in the hub module were selected as the DCM-related genes.

### Identification of hub genes

DCM-related genes, ferroptosis-related DEGs, and hypoxia-related DEGs were intersected to acquire intersection genes. Then, LASSO regression analysis was utilized to select the hub genes through the “glmnet” (version 4.1–1) package. Additionally, receiver operating characteristic (ROC) analysis was performed to evaluate the diagnostic value of hub genes in distinguishing DCM from healthy controls using the “pROC” (version 1.17.0.1) package. Area under the curve (AUC) was used to evaluate the diagnostic accuracy of hub genes.

### Immune cell infiltration analysis

The relative abundance of each immune cell was calculated using the single-sample GSEA (ssGSEA) algorithm. Signature gene sets marking various types of infiltrating immune cells were obtained from Charoentong’s study [12, 13], which covers a range of human immune cell subtypes, including activated CD8 T cell, activated dendritic cell, macrophages, monocyte, natural killer cell, and regulatory T cells. The difference in immune cell infiltration between DCM patients and healthy controls was compared using the Wilcox test. Furthermore, the correlation between differentially infiltrating immune cells and hub genes was explored.

### Construction of competing endogenous RNA (ceRNA) network

The miRNA-expressed dataset GSE112556 comprised 3 healthy controls and 3 heart tissue samples from DCM patients. The differentially expressed miRNAs (DEmiRNAs) with *p*-value <0.05 between DCM patients and controls were obtained. The Encyclopedia of RNA Interactomes (ENCORI,^5^) dataset was used to select hub gene-related miRNAs. Next, the predicted miRNAs were intersected with DEmiRNAs, and their intersection was taken to obtain miRNA-mRNA targeting pairs with negative regulatory relationships. Additionally, the miRNAs-related lncRNAs were attained through the ENCORI dataset. Next, the lncRNAs associated with the hub genes were defined as DCM-related lncRNAs by correlation analysis (*p*-value <0.05). Finally, the lncRNA-miRNA-mRNA network was constructed using the Cytoscape.

### Drug prediction and molecular docking

The Drug Gene Interaction Database (DGIdb,^6^) was utilized to obtain hub genes-related drugs. Molecular docking technology is to place small molecules (ligands) in the binding region of macromolecular targets (receptors) by computer simulation, and predict the binding energy (binding affinity) and binding mode (conformation) of the two by calculating physical and chemical parameters, and then identifies the lowest-energy conformations of ligand-receptor binding. Low binding energy is the basis of stable binding between molecules. The 3D structures of target proteins (receptors) and drugs (ligands) were downloaded from the RCSB PDB^7^ and the PubChem database^8^, respectively. Subsequently, proteins and drug molecules were hydrogenated and other pretreated in AutoDockTools, followed by molecular docking. In the docking results, binding energy was used as a reference to screen the most active ligand molecules and target genes. Binding energy <0 means that the ligand and receptor can bind spontaneously. The smaller the binding energy, the more stable the binding between ligand and receptor. Binding energy less than −5.0 kJ/mol (Note: −5.0 kJ/mol = −1.19423 kcal/mol) is the basis for screening candidate targets of active ingredients/drugs. Finally, the molecular docking results were visualized by the PyMol software.

### *In vitro* verification

The human cardiomyocyte AC16 was purchased from Wuhan Pricella Biotechnology Co., Ltd. Doxorubicin (DOX; 0.3 µM) was used to stimulate AC16 cells at 37 °C in a humidified atmosphere of 5% CO2 for 24 h to induce cardiomyocyte injury model. ATrizol (TIANGEN, DP424), FastQuant cDNA first strand synthesis (TIANGEN, KR106) and SuperReal PreMix Plus (SYBR Green) (TIANGEN, FP205) kits were used for RNA extraction, reverse transcription and RT-qPCR, respectively. The hub gene TGM2 was selected for knockdown experiments, and the expression of TGM2 was detected by RT-qPCR and western blotting. The sequence of siRNA is shown in Supplementary Table S1. According to the manufacturer’s instructions, apoptotic cells were evaluated using the Annexin V-FITC/PI apoptosis detection kit (Solarbio, CA1020). In addition, immunofluorescence staining was performed on cardiomyocytes in each group. After fixing and permeabilizing cells, incubate them with mouse anti-α-actinin monoclonal antibody (Biodragon, BD-PM3612) and rabbit anti-troponin T-C polyclonal antibody (Biodragon, BD-PT5362), followed by fluorescently labeled goat anti-mouse IgG (red, Biodragon, BD9276) and goat anti-rabbit IgG (green, Bioss, bs-0295G-FITC). Finally, stain the nuclei with DAPI and observe under a fluorescence microscope. In addition, the dual-luciferase reporter assay was used to investigate the association between hsa-miR-296-5p and TGM2.

## Results

### Identification of ferroptosis- and hypoxia-related DEGs and functional enrichment analysis

**FIGURE 1:**
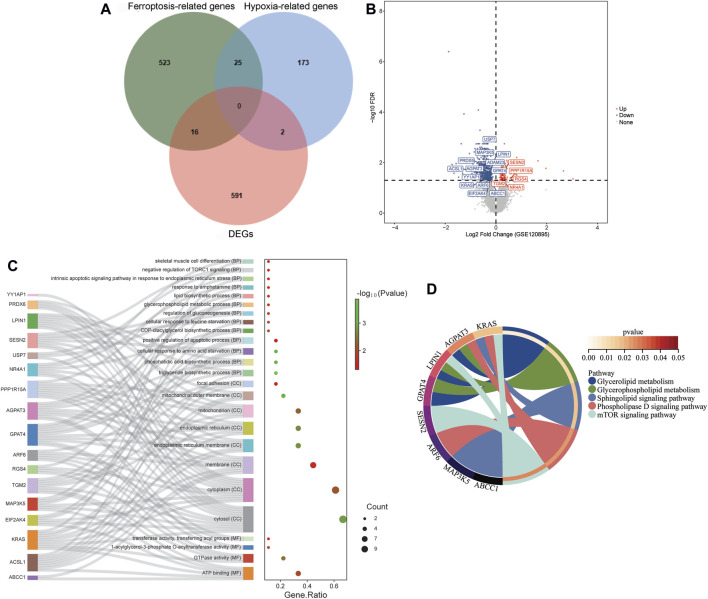
Identification of ferroptosis- and hypoxia-related DEGs. **(A)** Venn plot among ferroptosis-related genes, hypoxia-related genes, and DEGs in the GSE120895 dataset. **(B)** The volcano of ferroptosis- and hypoxia-related DEGs. **(C,D)** GO **(C)** and KEGG **(D)** analysis of ferroptosis- and hypoxia-related DEGs.

A total of 609 DEGs were identified in DCM patients compared to healthy controls in the GSE120895 dataset, including 102 up- and 507 down-regulated DEGs. Then, 564 ferroptosis-related genes, 200 hypoxia-related genes, and 609 DEGs were intersected (Figure 1A). Finally, 16 ferroptosis-related DEGs (SESN2, AGPAT3, MAP3K5, EIF2AK4, PRDX6, NR4A1, ARF6, KRAS, ABCC1, RGS4, LPIN1, YY1AP1, USP7, GPAT4, ACSL1, and ADAM23) and 2 hypoxia-related DEGs (PPP1R15A and TGM2) were acquired (Figure 1B).

In the GO:BP terms, these 18 genes were significantly enriched in triglyceride biosynthetic process, phosphatidic acid biosynthetic process and cellular response to amino acid starvation (Figure 1C). In the GO:CC terms, these 18 genes significantly enriched in cytosol, cytoplasm and membrane (Figure 1C). In the GO:MF terms, these 18 genes were significantly enriched in ATP binding, GTPase activity and 1-acylglycerol-3-phosphate O-acyltransferase activity (Figure 1C). The KEGG results showed that the DEGs were significantly enriched in mTOR signaling pathway, Glycerophospholipid metabolism and Glycerolipid metabolism (Figure 1D).

### Identification of DCM-related genes

**FIGURE 2:**
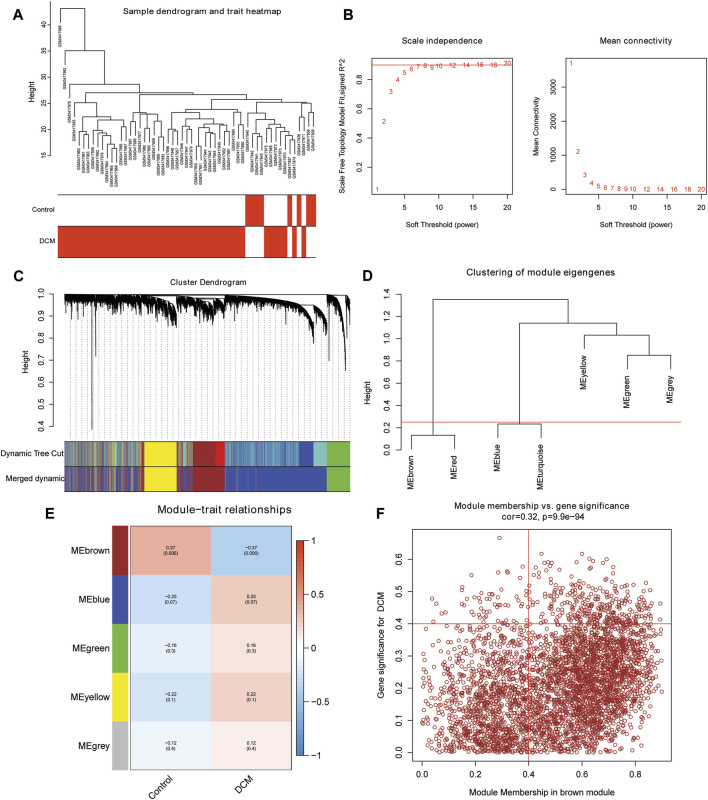
WGCNA. **(A)** Clustering dendrogram of all samples in the GSE120895 dataset. **(B)** Scale independence and mean connectivity analysis. **(C)** Clustering dendrogram of genes. **(D)** Clustering of module eigengenes. **(E)** The correlation between modules and groups. **(F)** The scatterplot of MM and GS in the brown module.

The GSE120895 dataset was used to carry out WGCNA. All samples were clustered, and the sample clustering tree is displayed in Figure 2A. Then, the soft threshold was set as 6 to construct the scale-free network (Figure 2B). The minimum number of genes in the module was set to 500, and 7 modules were attained (Figure 2C). Next, the brown and red modules were merged, as were the blue and turquoise modules (Figure 2D). Finally, 5 modules were identified. Subsequently, we found that the brown module was significantly correlated with DCM (Figure 2E). Then, a total of 315 DCM-related genes with |GS| >0.4 & |MM| >0.4 were acquired (Figure 2F).

### Identification of hub genes

**FIGURE 3:**
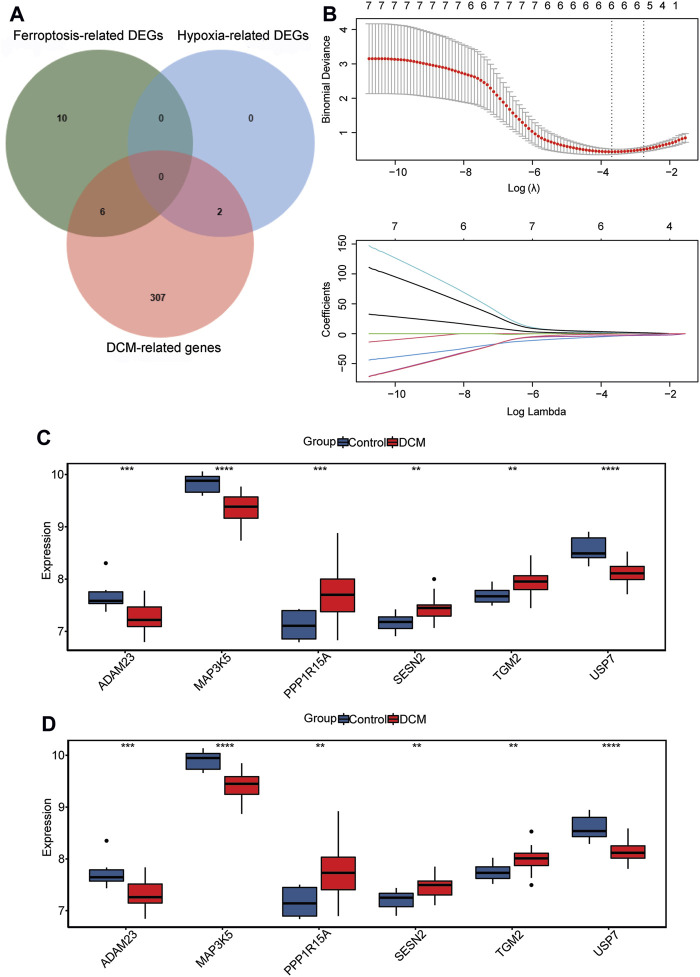
Identification of hub genes. **(A)** Venn plot among DCM-related genes, ferroptosis-related DEGs, and hypoxia-related DEGs. **(B)** LASSO regression analysis is used to identify hub genes. **(C,D)** The expression levels of hub genes between healthy controls and DCM patients in the GSE120895 **(C)** and GSE17800 datasets **(D)**.

**FIGURE 4:**
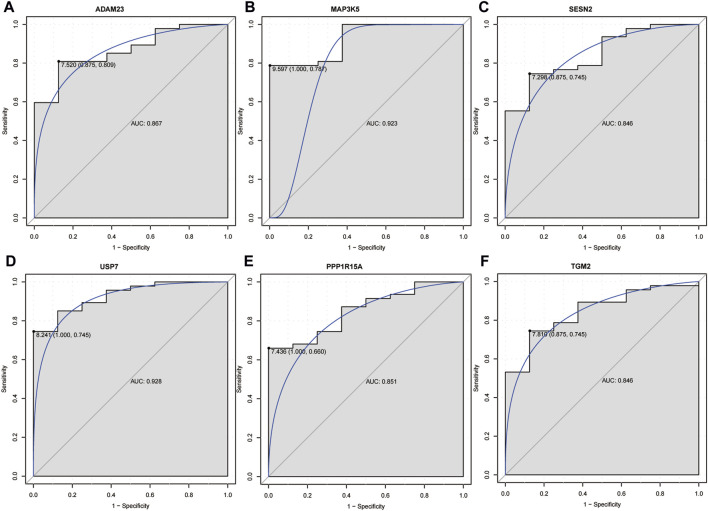
The ROC analysis of ADAM23 **(A)**, MAP3K5 **(B)**, SESN2 **(C)**, USP7 **(D)**, PPP1R15A **(E)**, and TGM2 **(F)** in the GSE120895 dataset. In ROC analysis, the greater the AUC, the higher the diagnostic accuracy. The AUC value of 0.8–0.9 indicated very good diagnostic accuracy, and 0.9–0.1 indicated excellent diagnostic accuracy.

The 315 DCM-related genes, 16 ferroptosis-related DEGs, and 2 hypoxia-related DEGs were intersected, and 8 intersection genes were acquired (Figure 3A). Subsequently, 6 hub genes (PPP1R15A and TGM2 for hypoxia and MAP3K5, USP7, SESN2, and ADAM23 for ferroptosis) were identified using LASSO regression analysis (Figure 3B). The expression levels of ADAM23, MAP3K5, and USP7 were significantly decreased in DCM patients, while the expression levels of PPP1R15A, TGM2, and SESN2 were significantly increased in DCM patients in both GSE120895 and GSE17800 datasets (Figures 3C,D). Additionally, ROC analysis showed that the AUC values of 6 hub genes were all more than 0.8 in both the GSE120895 and GSE17800 datasets (Figure 4; Supplementary Figure S1). Furthermore, a diagnostic model for DCM was constructed via LASSO regression based on the 6 hub genes. Results showed that the AUC of the model exceeded 0.9 in both the GSE120895 (Supplementary Figure S1A) and GSE17800 (Supplementary Figure S1B) datasets.

### Immune cell infiltration analysis

**FIGURE 5:**
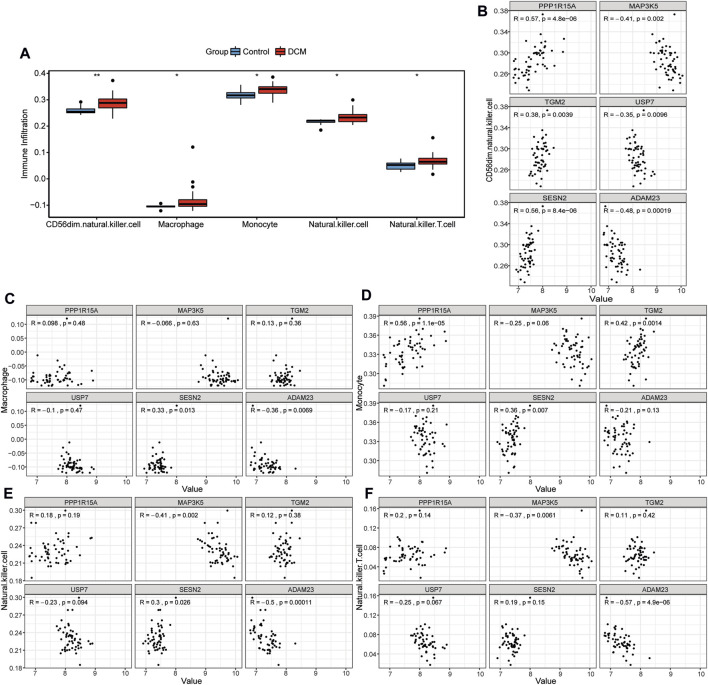
Immune cell infiltration analysis. **(A)** The box plot shows the difference in immune infiltration between DCM and healthy controls. **(B–F)** The correlation between hub genes and CD56dim NK cells **(B)**, macrophages **(C)**, monocytes **(D)**, NK cells **(E)**, and NK T cells **(F)** respectively.

Immune cell infiltration analysis results showed that CD56dim natural killer (NK) cells, macrophages, monocytes, NK cells, and NK T cells were significantly highly infiltrated in DCM patients in the GSE120895 dataset (Figure 5A). Additionally, the correlation between differentially infiltrating immune cells and hub genes was explored. CD56dim NK cells were significantly correlated with 6 hub genes (Figure 5B). Macrophages were significantly associated with SESN2 and ADAM23 (Figure 5C). Monocytes showed significant correlations with PPP1R15A, TGM2, and SESN2 (Figure 5D). NK cells and NK T cells were significantly correlated with MAP3K5 and ADAM23 (Figures 5E,F). Additionally, NK cells exhibited a significant correlation with SESN2.

### Construction of ceRNA network

**FIGURE 6:**
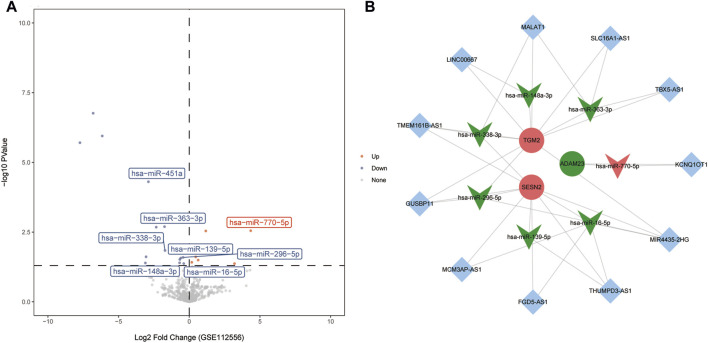
Construction of ceRNA network. **(A)** The volcano plot of DEmiRNAs in the GSE112556 dataset. **(B)** A ceRNA network in DCM. The rhombus represented lncRNA, the V type denoted miRNA, and the circle indicated mRNA. Red and green indicated upregulation and downregulation, respectively. The lines between nodes indicate predicted targeted regulatory relationships.

A total of 21 DEmiRNAs were acquired in the GSE112556 dataset, including 7 up- and 14 down-regulated DEmiRNAs (Figure 6A). Then, 11 miRNA-mRNA negative regulatory relationship pairs were obtained, including 8 DEmiRNAs. Subsequently, 11 lncRNAs were identified that interacted with 7 DEmiRNAs (hsa-miR-770-5p, hsa-miR-16-5p, hsa-miR-139-5p, hsa-miR-296-5p, hsa-miR-338-3p, hsa-miR-148a-3p, and hsa-miR-363-3p) and 3 hub genes (ADAM23, SESN2, and TGM2). Finally, the ceRNA network, including 11 lncRNAs, 7 miRNAs, and 3 mRNAs, was constructed (Figure 6B).

### Drug prediction and molecular docking

**FIGURE 7:**
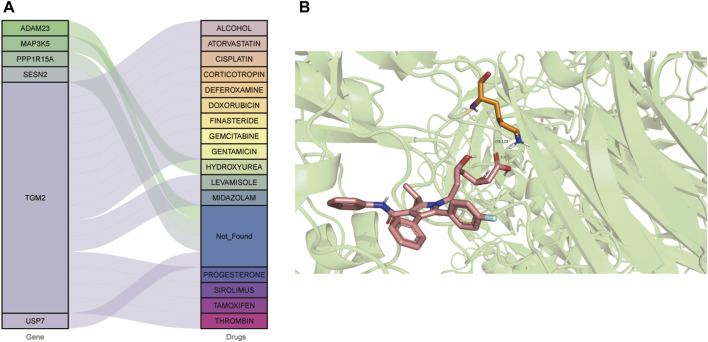
Drug prediction and molecular docking. **(A)** Drug prediction for hub genes. **(B)** The molecular docking between TGM2 and atorvastatin.

A total of 16 drugs were obtained (Figure 7A). Among these, 1 drug may target the MAP3K5 gene, 15 drugs may target the TGM2 gene, and the remaining genes were not matched to drugs. Literature retrieval revealed that atorvastatin, a drug associated with TGM2, has significant benefits in treating DCM, and low-dose use significantly reduces levels of inflammatory cytokines and uric acid, improves hemodynamic parameters, and increases the 5-year survival rate of DCM patients [14, 15]. Subsequently, molecular docking between atorvastatin and TGM2 was performed. Molecular docking between atorvastatin and TGM2 was performed. The binding energy was −2.79 kcal/mol by binding LYS-173 with 1 hydrogen bond (Figure 7B).

### *In vitro* verification of the hub gene TGM2

**FIGURE 8:**
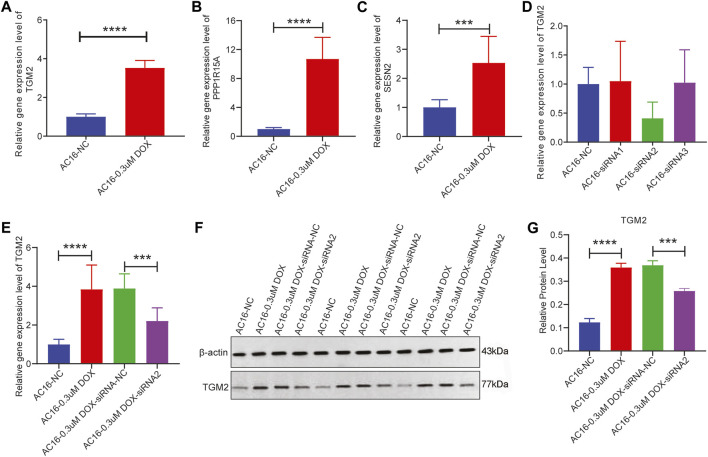
The expression of TGM2 in the DOX-induced AC16 cardiomyocyte injury model. **(A–C)** mRNA expression levels of TGM2, PPP1R15A, and SESN2 in DOX-induced AC16 cardiomyocyte injury. **(D)** The knockdown effect of 3 interference plasmids on TGM2 was detected by RT-qPCR. **(E)** mRNA expression levels of TGM2 in AC16-NC, AC16-0.3uM DOX, AC16-0.3uM DOX-siRNA-NC and AC16-0.3uM DOX-siRNA2 cells were detected by RT-qPCR. **(F)** The expression bands of TGM2 protein in AC16-NC, AC16-0.3uM DOX, AC16-0.3uM DOX-siRNA-NC and AC16-0.3uM DOX-siRNA2 cells were detected by western blotting. **(G)** The relative expression levels of TGM2 protein in AC16-NC, AC16-0.3uM DOX, AC16-0.3uM DOX-siRNA-NC and AC16-0.3uM DOX-siRNA2 cells.

The expression of the highly expressed hub genes (TGM2, PPP1R15A, and SESN2) in the DCM group was verified through RT-qPCR in AC16 cells treated with DOX. Compared with the control group, the expression levels of TGM2, PPP1R15A, and SESN2 in the DOX group were significantly increased (Figures 8A–C), which was consistent with the expression trend in the public database. Hub gene TGM2, a potential diagnostic biomarker, is not only associated with immune cell infiltration but also may play a crucial regulatory role in DCM via the lncRNA-miRNA-TGM2 axis. Moreover, drug prediction revealed that atorvastatin, a drug associated with TGM2, has significant benefits in treating DCM. Therefore, TGM2 was selected for subsequent experiments.

**FIGURE 9:**
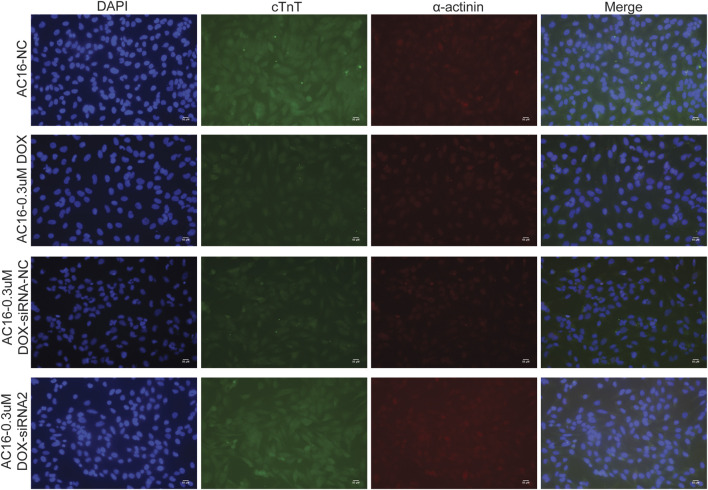
The influence of TGM2 on the expressions of cTnT and α-actinin was detected by immunofluorescence staining in DOX-induced AC16 cardiomyocyte injury.

**FIGURE 10:**
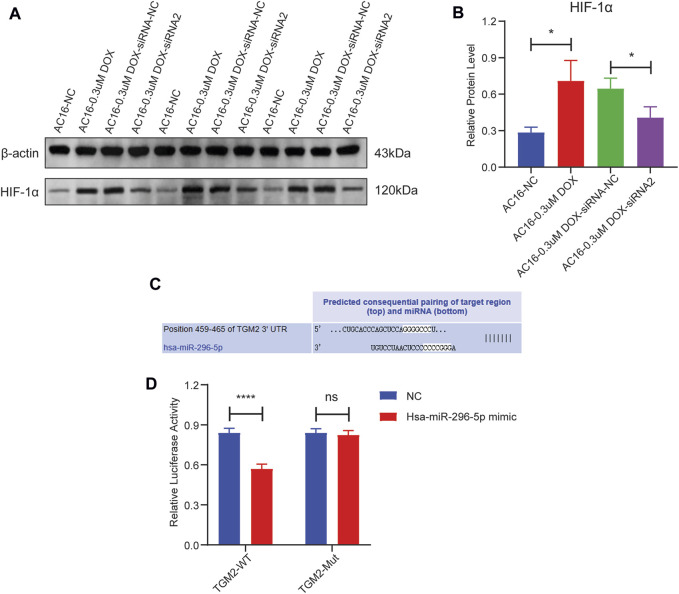
The effect of HIF-1α by TGM2 and validation of hsa-miR-296-5p targeting TGM2 via dual-luciferase reporter assay. **(A)** The expression bands of HIF-1α protein AC16-NC, AC16-0.3uM DOX, AC16-0.3uM DOX-siRNA-NC, AC16-0.3uM DOX-siRNA2. **(B)** The relative expression levels of HIF-1α protein in AC16-NC, AC16-0.3uM DOX, AC16-0.3uM DOX-siRNA-NC and AC16-0.3uM DOX-siRNA2 cells. **(C)** Bioinformatic prediction of the binding site between hsa-miR-296-5p and TGM2. **(D)** Validation of the targeted regulation of TGM2 by hsa-miR-296-5p using dual-luciferase reporter assay.

**FIGURE 11:**
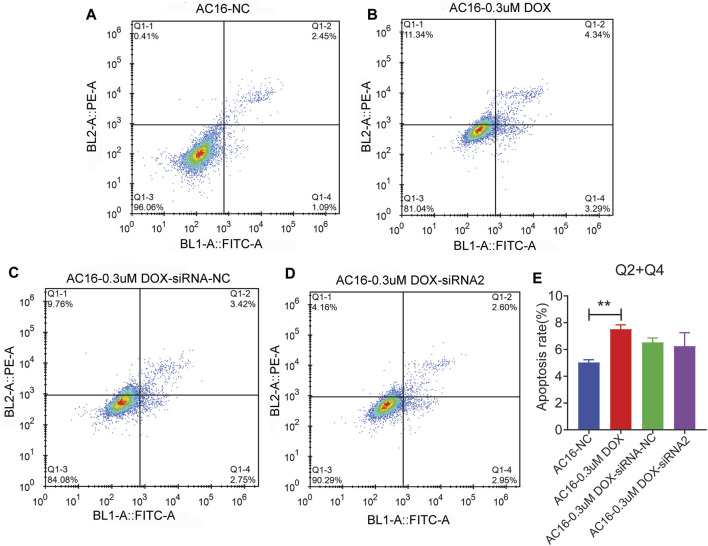
Flow cytometry was used to detect the effect of TGM2 on apoptosis of AC16 cardiomyocyte. **(A)** Flow cytometry was used to detect AC16-NC cell apoptosis. **(B)** Flow cytometry was used to detect AC16-0.3uM DOX cell apoptosis. **(C)** Flow cytometry was used to detect AC16-0.3uM DOX-siRNA-NC cell apoptosis. **(D)** Flow cytometry was used to detect AC16-0.3uM DOX-siRNA2 cell apoptosis. **(E)** Statistical histogram of apoptosis in flow cytometry.

TGM2 was knockdown via siRNA transfection, and TGM2 expression was quantified by RT-qPCR to identify effective siRNA targets. The results showed that the knockdown effect of target 2 was the best (Figure 8D). Therefore, siRNA-2 was selected for subsequent knockdown experiments. Subsequently, the expression of TGM2 in each group was detected by RT-qPCR and western blotting. The results showed that the expression of TGM2 was significantly increased after DOX treatment, while the expression of TGM2 was significantly decreased in the knockdown group (Figures 8E–G). Subsequently, immunofluorescence staining was performed to investigate the effects of TGM2 on cTnT and α-actinin (Figure 9). Compared with the control group, the expressions of α-actinin and cTnT in the DOX group were decreased. After knocking down TGM2, the expressions of α-actinin and cTnT were increased. Furthermore, after knockdown of TGM2, the expression level of HIF-1α was inhibited (Figures 10A,B). The complementary binding sites between hsa-miR-296-5p and the 3′ UTR of TGM2 were predicted through the ENCORI database (Figure 10C). Based on the psiCHECK 2 vector, we constructed TGM2-WT (wild-type) and TGM2-Mut (mutant) dual-luciferase reporter plasmids. Dual luciferase assay showed that hsa-miR-291-5p exerted its regulatory effect by directly binding to TGM2 (Figure 10D). In addition, flow cytometry results showed that TGM2 had no significant effect on the apoptosis of AC16 cells (Figures 11A–E).

## Discussion

DCM is a cardiac disease that eventually leads to HF and sudden cardiac death [16]. Ferroptosis is regulated necrosis and plays a crucial role in cardiovascular disease. Cardiomyocyte ferroptosis is involved in the pathogenesis and progression of DCM, and inhibition of ferroptosis can improve DCM-related pathological phenotypes [17]. Interleukin 27 may exert a protective effect in DCM by inhibiting ferroptosis [18]. Furthermore, hypoxia is the major risk factor for cardiovascular diseases. Hypoxia is associated with DCM [9], and asprosin protects cardiomyocytes by enhancing cardiac mitochondrial function under hypoxia, reducing the risk of adverse cardiovascular events in DCM patients [19]. Hypoxia can also regulate ferroptosis under certain conditions [20]. This study screened and identified hypoxia- and ferroptosis-related hub genes via bioinformatics analysis, providing new insights for subsequent related research.

In this study, a total of 6 hub genes (MAP3K5, USP7, SESN2, ADAM23, PPP1R15A, and TGM2) were acquired in DCM patients. Mitogen-activated protein kinase kinase kinase 5 (MAP3K5), also known as apoptosis signal-regulating kinase 1 (ASK1), participates in regulating cell fate in HF and reperfusion injury [21, 22]. Its abnormal expression is related to DCM [23]. Ubiquitin-specific protease 7 (USP7) is a component of the ubiquitin-proteasome system and plays a role in cardiomyocyte injury [24, 25]. Increasing USP7 could enhance ferroptosis in myocardial I/R rats [26]. Sestrin 2 (SESN2) is a stress-inducible protein associated with various stress conditions [27]. SESN2 can regulate cardiomyopathy by modulating mitophagy and mitochondrial function [28]. A disintegrin and metalloprotease 23 (ADAM23) belong to the transmembrane protein family, and its expression is down-regulated in heart tissues in DCM patients [29]. Transglutaminase 2 (TGM2) is a potential risk gene related to DCM [30]. Inhibiting TGM2 can alleviate myocardial fibrosis after myocardial infarction [31]. However, Protein phosphatase 1 regulatory subunit 15A (PPP1R15A) has not been reported in DCM. In this study, these hub genes have good performance in distinguishing DCM patients from healthy controls, suggesting that these hub genes may serve as potential biomarkers for DCM.

cTnT, as an important component of cardiac troponin, is a specific regulatory protein of cardiomyocytes. It collaborates with troponin I and C to regulate the contraction and relaxation of the myocardium, playing a key role in the excitation-contraction coupling mechanism of cardiomyocytes [32]. α-actinin is an actin-binding protein that is abundant in muscle tissues, and its main function is to maintain the structural stability of actin filaments. In cardiomyocytes, it is involved in the formation of the Z-line of the sarcomere, which is crucial for maintaining the contractile function and structural integrity of cells [33]. Previous studies have shown that cTnT and α-actinin play an indispensable role in the occurrence and development of DCM [34–36]. In this study, we found that TGM2 affected the expressions of cTnT and α-actinin. This revealed a novel mechanism by which TGM2 participates in the regulation of cardiomyocytes function by influencing the expression of cardiac structural proteins cTnT and α-actinin, providing potential molecular targets for understanding the pathological process of DCM. As a core molecule in the hypoxic response, existing studies have confirmed that HIF-1α is involved in the regulation of cardiac hypertrophy and heart failure [37, 38]. In this study, we also found that TGM2 affected the expression of HIF-1α. This result confirms that TGM2 plays a significant role in the hypoxia pathway of DCM. HIF-1α has been confirmed in previous studies to be involved in the ferroptosis process by regulating genes related to iron metabolism and lipid peroxidation [39, 40]. This provides potential associative clues for TGM2 to possibly intervene in ferroptosis regulation. Based on this, it is worth further study to explore the specific role and mechanism of TGM2 in the process of ferroptosis.

The immune response and its regulatory mechanisms play an important role in the occurrence and development of DCM [41]. Higher infiltrating levels of CD56dim NK cells, macrophages, monocytes, NK cells, and NK T cells were observed in DCM patients compared to healthy controls in this study. Stettner et al. demonstrated that the number of NK cells is significantly decreased, whereas the frequency of NK T-like cells is significantly increased in peripheral blood patients with idiopathic DCM [42]. However, NK cell activity in blood samples in DCM patients is decreased [43]. Among cardiac resident immune cells, macrophages, comprising approximately 7% of nonmyocytes, regulate cardiac impulse conduction, promote angiogenesis and vascular development, and maintain mitochondrial homeostasis [44, 45]. In the clinical study, DCM patients with higher infiltration levels of macrophage exhibited worse outcomes [46]. In DCM patients, increased expression of myeloid differentiation factor-2 (MD-2) could promote monocyte chemotactic protein 1 (MCP-1) secretion and monocyte recruitment, resulting in the progression of DCM [47]. The implantation of bone marrow-derived mononuclear cells into the LV anterior wall significantly relieves LV remodeling in DCM rats [48]. The Pearson correlation analysis revealed a certain degree of correlation between these immune cells and hub genes. Therefore, we speculate that the functions of these immune cells in DCM may be regulated via hub genes, and the specific molecular mechanisms deserve further investigation.

To further explore the possible molecular mechanisms involved in hub genes, a ceRNA network was constructed. The ceRNA network comprising 3 hub genes, 7 miRNAs, and 11 lncRNAs was constructed. Previous studies have shown that the abnormal expressions of hsa-miR-770-5p [49, 50], hsa-miR-16-5p [51], hsa-miR-338-3p [52] and hsa-miR-363-3p [49, 50] are involved in regulating the development of DCM. LncRNA KCNQ1OT1 can participate in regulating myocardial ischemia/reperfusion injury in mice and mediate apoptosis of myocardial cells [53, 54]. Inhibition of lncRNA MIR4435-2HG improved infarction volume, ejection fraction, and cardiomyocyte apoptosis [55]. LncRNA FGD5-AS1 is abnormally expressed in DCM and is involved in the regulation of cardiomyocyte apoptosis and cardiac fibrosis [56, 57]. LncRNA MALAT1 can regulate the microvascular function after myocardial infarction in mice by modulating mitochondrial dynamics [58]. Moreover, it can also mediate high glucose-induced cardiomyocyte apoptosis through the RhoA/ROCK pathway [59]. Based on the previous studies mentioned above, we speculated that the KCNQ1OT1-hsa-miR-770-5p-ADAM23, MIR4435-2HG/FGD5-AS1-hsa-miR-16-5p-SESN2 and MALAT1-hsa-miR-338-3p-TGM2 axes identified in this study may play an important regulatory role in the progression of DCM. To date, no studies have been reported on hsa-miR-139-5p, hsa-miR-296-5p, and hsa-miR-148a-3p in DCM. In this study, hsa-miR-296-5p was selected for dual-luciferase reporter assay, and the results showed that it had a direct targeting binding relationship with TGM2. This suggests that hsa-miR-296-5p may exert its function in DCM by targeting and regulating TGM2, while its specific mechanism remains to be further explored.

TGM2 is a Ca^2+^-dependent transglutaminase that participates in apoptosis, cell survival, phagocytosis, cell adhesion and migration, and cell signaling [60]. Drug prediction results showed that atorvastatin, sirolimus, and thrombin were predicted to target TGM2. Atorvastatin improves left ventricular ejection fraction in DCM patients in the meta-analysis [14]. Sirolimus, also known as rapamycin, relieves oxidative stress, protects mitochondria, and restores energy homeostasis in DCM mice [61]. Additionally, sirolimus improves heart function in various cardiomyopathies, including alcoholic cardiomyopathy, cirrhotic cardiomyopathy, and hypertrophic cardiomyopathy [62–64]. Noteworthily, the mTOR (mammalian target of rapamycin) pathway was found to be enriched in our study. Wang et al. reported that inhibition of the AMPK/mTOR pathway reduces myocardial damage and attenuates diabetic cardiomyopathy [65]. Thrombin expression was significantly up-regulated in DCM patients compared to healthy controls through immunohistochemical [66]. After inhibition of thrombin, left ventricular function and poor outcomes in DCM mice were significantly improved [66]. From the perspective of transformation, atorvastatin and sirolimus have been clinically used for cardiovascular indications, and if their efficacy through TGM2 targeted therapy is confirmed, it may contribute to their use in DCM. Although the regulation of thrombin has shown promising application prospects in preclinical studies, its safety and efficacy characteristics still need to be strictly evaluated in cardiomyopathy. Overall, these predicted drugs offer testable candidates for future translational studies aimed at DCM therapy.

## Conclusion

Through bioinformatical analysis, 6 hub genes (PPP1R15A, TGM2, MAP3K5, USP7, SESN2, and ADAM2) were identified, which may be potential diagnostic biomarkers for DCM. Preliminary *in vitro* verification showed that TGM2 was up-regulated in DOX-induced AC16 cardiomyocyte injury. After knocking down TGM2, the expressions of α-actinin and cTnT were increased, and the expression level of HIF-1α was inhibited. Dual luciferase assay showed that hsa-miR-291-5p exerted its regulatory effect by directly binding to TGM2. Our findings may facilitate understanding the potential pathogenesis of DCM and provide new insights for the treatment of DCM.

## Data Availability

The datasets presented in this study can be found in the GEO database (http://www.ncbi.nlm.nih.gov/geo). The accession numbers are GSE120895, GSE17800, and GSE112556, respectively.

## Footnotes

1
https://www.ncbi.nlm.nih.gov/geo/

2
http://www.zhounan.org/ferrdb/current/

3
https://www.gsea-msigdb.org/gsea/msigdb/

4
https://david.ncifcrf.gov/

5
http://starbase.sysu.edu.cn/index.php

6
https://dgidb.org/

7
http://www.rcsb.org/pdb/home/home.do

8
https://pubchem.ncbi.nlm.nih.gov/
