# Supplementary material for: Identification of potential hub genes related to ferroptosis and hypoxia in dilated cardiomyopathy: a bioinformatic analysis with preliminary experimental validation
Source: Exp Biol Med (Maywood). 2026 Mar 2;251:10709. doi: 10.3389/ebm.2026.10709 (PMC12989450; doi:10.3389/ebm.2026.10709)
Supplement: Supplementary file 1 [file Table1.docx]

**Table S1 Sequence information of siRNA**

| **Target Name Primer** | |
| --- | --- |
| hTGM2 si-1 sense | ACAGCAACCUUCUCAUCGAGUTT |
| hTGM2 si-1 antisense | ACUCGAUGAGAAGGUUGCUGUTT |
| hTGM2 si-2 sense | GGCUGAAGAUCAGCACUAATT |
| hTGM2 si-2 antisense | UUAGUGCUGAUCUUCAGCCTT |
| hTGM2 si-3 sense | CCACCCACCAUAUUGUUUGAUTT |
| hTGM2 si-3 antisense | AUCAAACAAUAUGGUGGGUGGTT |
| d-NC sense | UUCUCCGAACGAGUCACGUTT |
| d-NC antisense | ACGUGACUCGUUCGGAGAATT |
