# Supplementary figures and images for: Identification of potential hub genes related to ferroptosis and hypoxia in dilated cardiomyopathy: a bioinformatic analysis with preliminary experimental validation
Source: Exp Biol Med (Maywood). 2026 Mar 2;251:10709. doi: 10.3389/ebm.2026.10709 (PMC12989450; doi:10.3389/ebm.2026.10709)

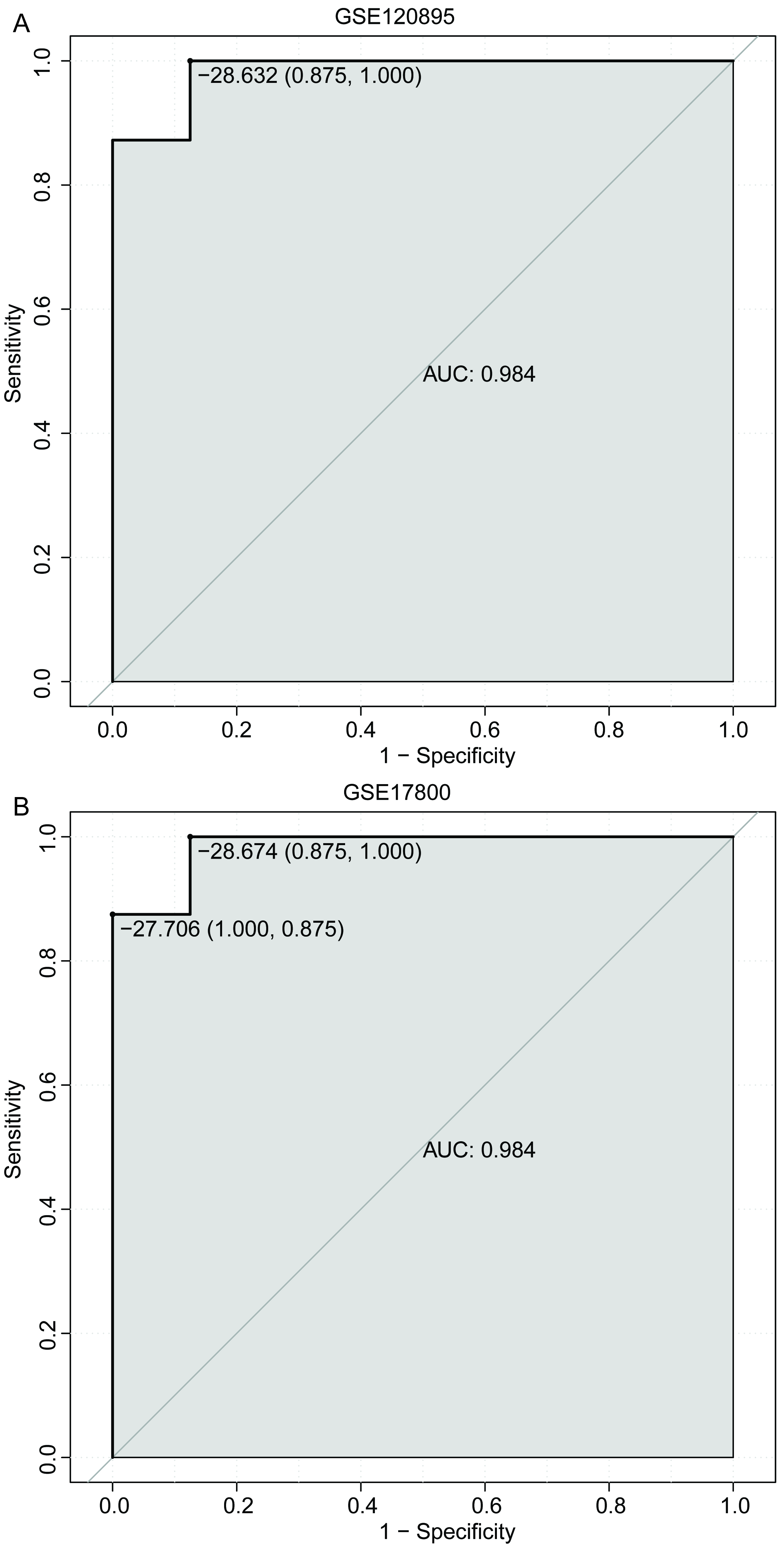

Supplement: Supplementary file 2 [file Image2.tif]

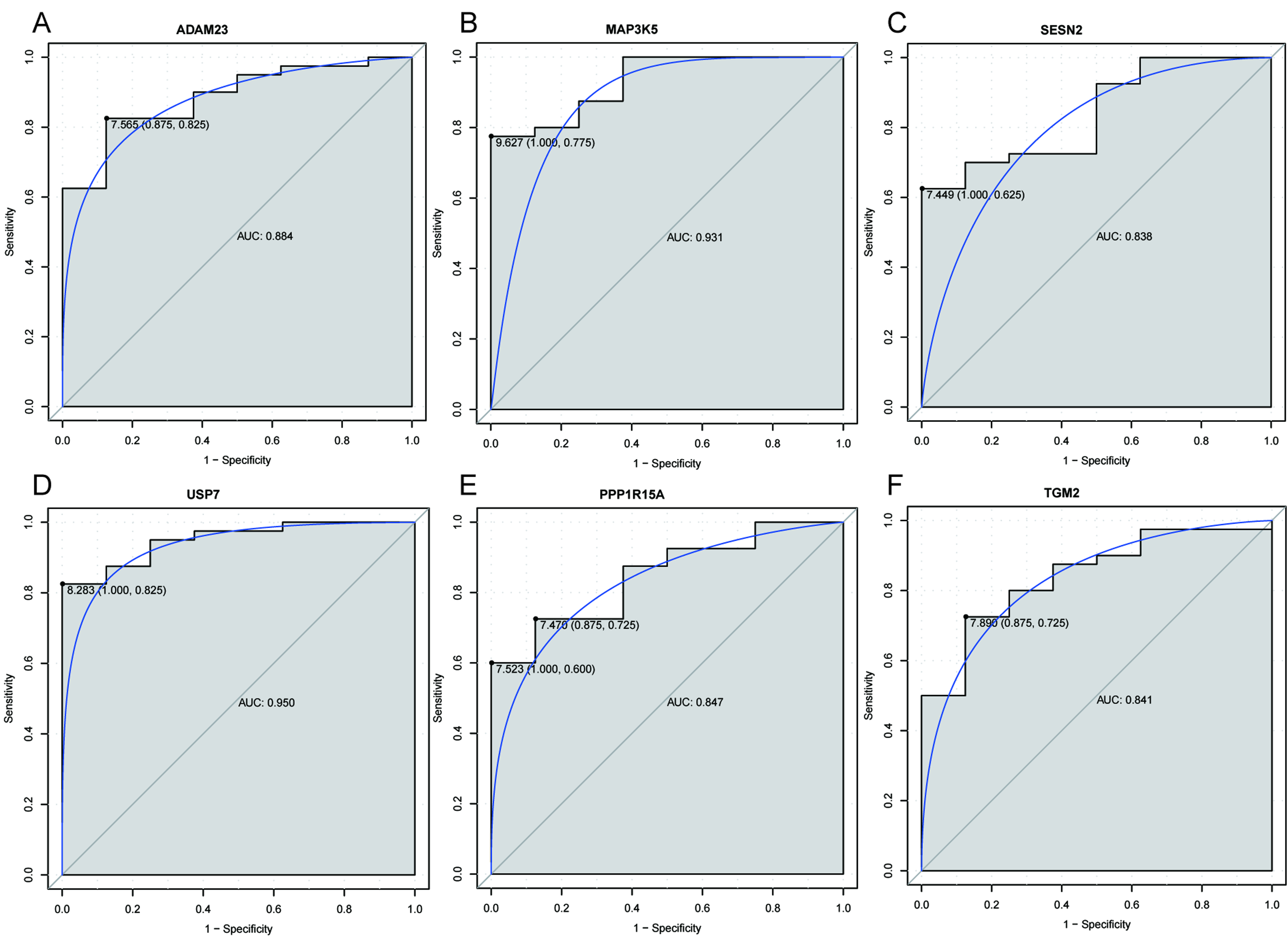

Supplement: Supplementary file 3 [file Image1.tif]
